# Quality of life in children with familial Mediterranean fever

**DOI:** 10.1186/1546-0096-13-S1-P130

**Published:** 2015-09-28

**Authors:** Ö Öztürk, S Yüksel, E Karadağlı, H Evrengül, B Özhan, M Tuğrul, O Kuzucu, E Uçar

**Affiliations:** 1 Pamukkale University, School of Medicine, Child and Adolescent Psychiatry, Denizli, Turkey; 2 Pamukkale University, School of Medicine, Pediatric Rheumatology, Denizli, Turkey; 3 Pamukkale University, School of Medicine, Pediatrics, Denizli, Turkey; 4 Pamukkale University, School of Medicine, Medical Student, Denizli, Turkey

## Objective

Familial Mediterranean fever (FMF) is a lifelong disorder, characterized by self-limited and recurrent attacks of fever and polyserositis. It is known that many chronic diseases have a negative effect on quality of life (QoL) multidimensionally. In our study, we aimed to assess the quality of life and psychological factors (anxiety and depression) in children with FMF.

## Method

A prospective cross-sectional study was conducted between September 2013 and September 2014. A total of 70 consecutive children with FMF who were diagnosed according to the Tel-Hashomer and Yalçınkaya criteria during the attack free period and 70 healthy children who were matched in terms of age and sex were enrolled. The Pediatric Quality of Life Inventory 4.0 (PedsQLTM 4.0), Child Depression Inventory (CDI) and Screen for Child Anxiety and Related Disorders (SCARED) were used for the psychosocial assessment.

## Results

Mean age of the patients (27 girls and 43 boys) was 11±3 years. The physical health, psychosocial health and total summary scores of the children with FMF were significantly lower than healthy children. In terms of sub dimension of psychosocial health, in the children with FMF, emotional functioning and school functioning domains’ scores were significantly lower than healthy children. Depression and anxiety scores were higher in the children with FMF than in healthy children.

**Table 1:** 

		Children with FMF	Healthy Children	p
		**Mean ±SD**	**Mean ±SD**	

**Physical Health**		77,7 ± 13,19	88,93 ± 10,17	<0.001*
**Psychosocial Health**		77,43 ± 13,04	87,26 ± 5,18	<0.001*

	**Social Functioning**	90,14 ± 12,94	89,93 ± 7,54	**0.093**
	
	**Emotional Functioning**	73,29 ± 19,85	88,43 ± 6,23	<0.001*
	
	**School Functioning**	68,86 ± 16,07	83,43 ± 13,47	<0.001*

**Total Summary Score**		77,5 ± 11,26	87,68 ± 4,68	<0.001*

**Depression Scores**		15,43 ± 5,75	9,87 ± 2,83	<0.001*

**Anxiety Scores**		22,9 ± 12,63	17,21 ± 7	0.004*

Independent samples t test, *statistical significance

## Conclusion

We found that the children with FMF have high level of depression and poorer QoL. FMF is a life-long disorder that has not only physical but also psychosocial impairments for the affected children. Therefore a biopsychosocial approach should be essential to treatment of the FMF.

